# Impact of climate change driven freshening, warming, and ocean acidification on the cellular metabolism of Atlantic Cod (*Gadus morhua*)

**DOI:** 10.1038/s41598-025-21597-z

**Published:** 2025-10-23

**Authors:** Peter Thor, Diana Perry

**Affiliations:** Department of Aquatic Resources (SLU Aqua), Institute of Marine Research, Swedish University of Agricultural Sciences, Turistgatan 5, Lysekil, 453 30 Sweden

**Keywords:** Atlantic cod, Climate change, Freshening, Warming, Ocean acidification, Metabolomics, Metabolic changes, Amino acid metabolism, TCA cycle, Glutamate metabolism, Biochemistry, Climate sciences, Ecology, Ecology, Environmental sciences, Ocean sciences

## Abstract

Climate change is causing increasing sea surface temperature, ocean acidification and, in near shore waters, freshening. We investigated the metabolic effects of all three and their combination in Atlantic cod from the Skagerrak (eastern North Sea) by measuring concentration changes of a wide range of metabolites involved in energy production in the liver and muscles. Liver metabolism was more strongly affected than muscle, reflecting its central regulatory role. Most amino acid concentrations declined in both tissues across all treatments, and metabolomic pathway analysis revealed significant enrichment in ten metabolic pathways. This suggests enhanced amino acid metabolism in a climate change future. Warming and ocean acidification induced increased liver concentrations of lactate, glucose and fructose 1,6-bisphosphate indicating that gluconeogenesis will increase to meet increased production of enzymes to counter future stress. The molar contribution of glutamine to the total change in liver amino acids constituted 49%, 16% and 29% under warming, ocean acidification and their combination accentuating its importance in energy production also under future climate change. We observed contrasting responses in AMP, ADP, and NAD^+^ concentrations between warming and acidification suggesting possible antagonistic effects. Our findings demonstrate significant and complex metabolic responses to future climate stress in Atlantic cod in northern European waters.

**Supplementary Information:**

The online version contains supplementary material available at 10.1038/s41598-025-21597-z.

## Introduction

In the ocean, the strongest driver of change related to climate change is warming of the upper water column. Since the rate of all biological processes increase with temperature, the warming of the upper ocean permeates the physiology of all organisms living here. These changes reverberate through the marine ecosystem creating change on all imaginable levels of biology, from specific physiological processes within cells to the interplay of species in the food webs^1,2^. But warming is not the only consequence of climate change in the marine realm. Increasing atmospheric CO_2_ partial pressure causes an influx of CO_2_ into the upper ocean leading to acidification from increasing concentrations of carbonic acid^3^. Moreover, in the shelf seas, increasing precipitation increases the input of fresh water from land and this drives an overall decrease in salinity especially in nearshore and estuarine waters^4^. The North Sea is experiencing the brunt of these changes. Mean annual surface water temperature is increasing at a rate of 0.37 °C per decade^5^ and pH is decreasing 0.024 per decade^6^. Changes in salinity are connected to precipitation patterns and may vary considerably on short time scales. Nevertheless, many larger coastal areas experience longer term changes superimposed on these variations as a result of longer term changes in overall precipitation patterns, and in the Kattegat, the shallow sea between Denmark and Sweden, salinity decreases at a rate of 0.12 PSU per decade^7^, a decrease which is projected to continue^8^. Organisms living in these waters not only experience one or the other of these changes but are exposed to the full force of their combined impact.

Coastal waters of the eastern North Sea; the Kattegat and the Skagerrak, constitute important nursery grounds and habitats for young Atlantic cod^9–12^. While the offshore waters are influenced by oceanic variations in the North Atlantic^13^, nearshore waters are strongly influenced by river runoff and their shallowness increases the influence of variations in atmospheric temperature. It is in these waters where the cod experiences the most significant changes in temperature, salinity and pH during their lifetime^14^, and it is here climate change affects the cod stocks most strongly. High fishing pressure has weakened the North Sea cod stocks^15–17^, and future population developments depend on ever decreasing numbers of young cod; the very same young cod which have to endure detrimental physiological effects of the changing coastal environment or face earlier migration to deeper waters^18,19^. As a consequence, the negative impacts of climate change in coastal waters have become ever more influential on recruitment and the recovery potential of the stocks as a whole^20–22^. Hence, several studies have shown that the North Sea cod stock experience decreasing recruitment with increasing temperature^23,24^.

Empirical knowledge on individual survival, growth and reproduction is pivotal for predictions of population recruitment, growth and development. To evaluate the possible trajectories of populations, one must rely on experimental determination of these individual traits, and laboratory studies help elucidate the physiological tolerance of fish to the effects of environmental changes. However, the magnitude of individual traits are determined by many underlying metabolic processes. Thus, when environmental conditions change—not merely along a single variable, as is commonly investigated under laboratory settings, but across a suite of interacting factors, as occurs under climate change—these metabolic processes may be altered in multiple ways, thereby rendering the resulting trait responses difficult to predict. Understanding the underlying metabolic consequences of exposure to multiple aspects of climate change is therefore important if we are to predict impacts on fish stocks as ocean changes intensify. While some studies have shown that increasing temperature will induce fundamental changes in the cellular metabolism in zebrafish (*Danio rerio*), salmon (*Salmo salar*) and rainbow trout (*Oncorhynchus mykiss*)^25–27^, much less is known about the effects of ocean acidification or freshening, or the combination of all three.

In the present study, we investigated the metabolic effects of warming, freshening, ocean acidification and their combination in Atlantic cod from the Swedish west coast. Metabolic reactions to climate change are often estimated from measured rates of exchange of elements or molecules with the environment. In such studies, measurements of the intake of carbon, nitrogen or specific fatty acids or the respiratory demand for oxygen are used to calculate the overall rates at which acquisition of essential metabolites or energy take place. But the metabolic reaction may be more complex and may be more of a reconfiguration of the metabolic apparatus rather than a change in overall rates of exchange of elements. Therefore, we investigated the changes in concentrations of a wide range of metabolites involved in energy production to allow detection of possible changes in the multitude of biochemical processes, which constitute the full metabolism of the fish.

## Methods

The animals used and experimental setup and exposure are the same as those from a previously published study on the effects of freshening, warming and ocean acidification on respiration rate, biometrics and enzymatic activity related to oxidative stress^19^. However, all the metabolomics data and analyses presented here are unique and not previously published.

### Animals

Juvenile cod were collected during October 2020 on the Swedish west coast (58°13’2.2”N, 11°24’46.2”E) and transported to the laboratory at the Kristineberg Centre for Marine Research and Innovation. A total of 100 juvenile cod (24.3 ± 3.5 cm, 159.4 ± 72.6 g) were used for the experiment. All animal care and experimental procedures followed relevant guidelines and regulations, with approval from the Swedish Board of Agriculture’s ethical committee in Gothenburg (permit Dnr 83-2016/ Dnr 5.8.18–15787/2020). The fish were housed in tanks with flow-through filtered seawater pumped from a depth of 32 m from the ocean outside the laboratory, maintained on a 12 h:12 h light: dark cycle, and fed ad libitum with a mixture of shrimp and herring. The fish were held in acclimation at ambient conditions (13.2 °C ± 0.1 SD, 29.7 ± 4.5 SD salinity) for at least 5 days preceding the exposure. Prior to the start of the experimental period, all fish were randomly placed in one of 20 cylindrical tanks, each with a volume of 3800 L. The experimental exposure was run for four weeks.

### Experimental setup and exposure

The experimental design consisted of five treatments: control, freshening (decreased salinity), warming (increased temperature), ocean acidification (decreased pH), and All (decreased salinity, increased temperature and decreased pH), 20 fish per treatment^19^.

For the freshening treatment, freshwater was mixed into the seawater supply before entering the tanks, ensuring a stable and consistent salinity level. For the warming treatment, temperature was controlled centrally using the computer control at the marine research station. For the ocean acidification treatment, pure CO_2_ was bubbled into the tanks, with water circulation promoting mixing. A pH-stat feedback system (Aqua Medic, Bissendorf, Germany) was used to maintain low pH. In the All treatment, all three global climate change factors (freshening, warming, and ocean acidification) were applied simultaneously throughout the experiment.

Temperature, salinity and pH were measured 1–3 times per week in all tanks using a WTW Multi 3430 pH meter. In addition to the measurements taken for all tanks, one to two tanks were measured daily to ensure that conditions remained stable. This was deemed sufficient given that the temperature and salinity were controlled centrally at the marine research station, and within treatments the tanks varied only marginally from one another. Every week, filtered water samples (0.45 μm) were collected for analysis of total alkalinity (TA) using a TA05 plus/TW alpha plus system (SI Analytics, Mainz, Germany). Total scale pH (pH_TS_) was measured using a pH meter calibrated with TRIS and AMP buffer solutions and *p*CO_2_was calculated from the pH_TS_ and TA data. To avoid shocking the fish, for the warming, the temperature was increased by approximately 1 °C per day, reaching the target value by day 5 of the exposure period. Salinity and pH values were set at the beginning of the exposure period.

The mean temperature was 17.4 ± 0.6 °C in the warming treatment, 16.9 ± 0.6 °C in the All treatment and 13.0 ± 0.5 °C across all other treatments, representing present day levels, whereas mean salinity was 26.2 ± 0.7 PSU in the freshening treatment, 26.5 ± 2.2 PSU in the All treatment and 32.6 ± 0.7 PSU across all other treatments, representing present day levels [19]. Mean pH_TS_ was 7.79 ± 0.25 (*p*CO_2_ of 984 ± 720 µatm) in the ocean acidification treatment, 7.55 ± 0.29 (*p*CO_2_ of 1427 ± 785 µatm) in the All treatment, and 8.06 ± 0.2 (*p*CO_2_ of 404 ± 89 µatm) across all other treatments, representing present day levels (Table 1). Temperature, salinity and pH in the treatments with exposure to future levels differed significantly from that of present day levels (Student’s t-tests: *P* < 0.001). For a detailed description of the water chemistry conditions during the experiment, please see Perry et al. 2024^19^.

**Table 1:** Salinity (S), temperature (T), total alkalinity (A_T_), CO_2_ partial pressure (*p*CO_2_), and total scale pH (pH_TOT_) of measurements taken throughout the four week exposure period (mean values and standard deviations, SD). The table is modified from Perry et al. 2024^19^.

Treatment	S, PSU	T, °C	A_T_, µmol kg^− 1^	pCO_2_, µatm	pH_TOT_
	Mean (SD)	Mean (SD)	Mean (SD)	Mean (SD)	Mean (SD)
Control	33.0 (0.3)	12.8 (0.4)	2405 (63)	390 (88)	8.08 (0.07)
Freshening	26.2 (0.7)	13.2 (0.2)	2139 (229)	368 (86)	8.09 (0.08)
Warming	33.2 (0.3)	17.4 (0.6)	2379 (59)	454 (83)	8.02 (0.07)
Ocean acidification	31.7 (3.1)	12.9 (0.4)	2326 (183)	984 (720)	7.79 (0.25)
All	26.5 (2.2)	16.9 (0.6)	2144 (214)	1427 (785)	7.55 (0.29)

At the end of the exposure period, all fish were euthanised with a sharp blow to the head followed by exsanguination. After euthanisation, samples of liver and muscle tissue were collected from each fish. The muscle samples were taken from the dorsal tissue just beside the dorsal fin. All samples were flash frozen in liquid nitrogen and stored at -80 °C until further analysis. For the shipment to the metabolomics lab, sub-samples were cut from the original samples while still frozen, transferred to pre-weighted 2 mL centrifuge tubes and freeze dried in a ScanVac Coolsafe 110-4 (LaboGene A/S, Allerød, Denmark). The dried samples were then weighed while still frozen, placed in a dry shipper (Voyageur 12, Cryopal, Bussy Saint Georges, France) previously saturated with liquid nitrogen and shipped to Les Laboratoires Iso-BioKem, Rimouski, Canada for chemical analysis.

### Chemical analyses

Metabolites were extracted and quantified by Les Laboratoires IsoBioKem Inc. (Rimouski, Canada) following their established protocols see also^28^. Forty-nine target metabolites were quantified in a total of 180 samples of liver and muscle from the cod in the five treatments. Positively and negatively charged metabolites were analysed separately.

At the lab, samples were initially homogenized for 30 s using a Precellys 24 (Bertin corp.) tissue homogenizer and stored at -80 °C until extraction of the metabolites. At the start of extraction, lipids were removed from the homogenised samples by filtration through Agilent Captiva EMR–Lipid filters using a trifluoroethanol-water solvent. The extracts were then vacuum dried (SpeedVac Concentrator Savant SPD2010, Thermo Scientific) and reconstituted in acetonitrile-water and subsamples were taken for the positive and negative analyses. Internal standards (valine-d_8_ for the positive analysis and pyrovate-d_3_ for the negative analysis) were added prior to extractions to allow compensation for any variations in extraction efficiency among samples.

For quantification of the targeted metabolites, the extracts were injected into a high-performance liquid chromatography system (HPLC 1260 Infinity II, Agilent Technologies, Santa Clara, CA, USA) coupled to a mass spectrometer (6470B Triple Quad, Agilent Technologies) using an InfinityLab Poroshell 120 HILIC-Z, 2.7 μm-column. HPLC-MS parameters were adjusted differently to fit the requirements of the analyses of the positive and negative metabolites.

The absolute quantification of metabolites in the extracts (ng mL^− 1^) was accomplished with the MassHunter QQQ quantitative (Quant-my-Way) software (Agilent Technologies) using standard curves for each metabolite with compensation for extraction variations detected by variations in the internal standards in every sample. Standards were produced at Les Laboratoires IsoBioKem Inc. Tissue metabolite concentrations (ng mg^− 1^) were calculated by dividing the extract concentration (ng mL^− 1^) with the sample weight (mg) and multiplying with the extract volume (mL).

### Data analyses

Of the 49 target metabolites, the concentrations of six metabolites in the liver samples and three metabolites in the muscle samples were below detection limits for all samples, and these were therefore not included in the analyses. These were acetyl-coenzyme A, ATP, NADH, NADPH, octopine, and phosphoenolpyruvate in the liver samples, and acetyl-coenzyme A, NADH and octopine in the muscle samples. In two liver samples from the salinity treatment, most of the metabolites showed a high number of missing values, so both samples were excluded entirely, and one liver sample was detected as an outlier in the PLS-DA analysis and therefore also excluded.

Prior to analyses, statistical outliers were identified as values falling outside ± 1.5 IQR (Interquartile Range) within each treatment and removed from the dataset. Missing data were then imputed using the MetImp 1.2 online software with the “missing not random, GSimp”-protocol^29,30^.

Differences in tissue concentrations of all metabolites between treatments and the control were analysed by 1-factor ANOVA followed by Dunnett’s post-hoc test. Differences in the multivariate variation in concentrations of metabolites belonging to the glycolysis, the TCA cycle (including metabolites carrying energy or reducing power) and proteogenic amino acids among the five treatments were analysed by 1-factor PERMANOVA in Primer 6+^31^. For this, data were first square root-transformed, resemblance matrices constructed using Euclidian distances, and the type III model was run using unrestricted permutations of raw data.

All analyses of changes in the metabolome were accomplished using the MetaboAnalyst 6.0 metabolomics analysis online software^32^. All metabolite concentrations were auto-scaled (mean-centred and divided by the standard deviation of each variable) and log_10_-transformed. The variation in the overall structure of metabolite concentrations in samples within and between the single treatments and the control were tested by partial least square discriminant analysis (PLS-DA). This analysis also identifies the importance of every metabolite to the differences between the treatment and the control. Enriched metabolic pathways (i.e. pathways where a number of functionally related metabolites change their concentrations) were identified by Metabolite Set Enrichment Analysis (MSEA) by pairwise comparison of metabolite concentrations between each treatment and the control. For this, changes in metabolite concentrations were screened against the Kyoto Encyclopaedia of Genes and Genomes (KEGG). Metabolomic Pathway Analysis (MetPA) was used to detect enriched pathways with significantly altered concentrations of metabolites with high pathway impact (metabolites at important positions within the pathway and hence likely to have significant impact on the pathways). The zebrafish, *Danio rerio*, metabolite library was chosen as reference. This is the taxonomically nearest fish for which a library exists. For the MetPA, we used the relative betweenness centrality as the importance measure for each metabolite.

Previous investigations of metabolic rate and oxidative stress enzyme activity in the same individuals found two distinct ecotypes that reacted differently in the freshening and All treatments^19^. We therefore tested for differences between these two groups of individuals and found significant differences only in very few metabolites (ANOVA) and no significant differences when testing the multivariate difference between treatments and control (PERMANOVA). We therefore pooled both ecotypes to avoid low statistical power.

## Results

### Metabolites

In the control, concentrations of glucose and lactate were the highest of the metabolites involved in the glycolysis, and concentrations of malate and succinate were the highest of metabolites involved in the TCA cycle (Table 2). For proteogenic amino acids, concentrations of alanine, glutamate and glycine were the highest. Comparing the two tissues, concentrations of metabolites carrying energy or reducing power and metabolites involved in glycolysis were higher in the muscles than in the liver (Table 2). Concentrations of metabolites involved in the TCA cycle were also higher in muscles than the liver. Of the proteogenic amino acids, concentrations of alanine, glycine and histidine were higher in the muscles, whereas glutamate concentrations were higher in the liver (Table 2).

**Table 2:** Mean ± SD concentrations of metabolites in the liver and muscles of Atlantic cod in the control treatment. *bd* = below detection limit.

Main function	Metabolite	Concentration, nmol g^− 1^	
		Liver	Muscles
Carrying energy or reducing power	AMP	87.5 ± 36.2	376 ± 250
	ADP	26.6 ± 12.7	1 547 ± 479
	ATP	*bd*	1 622 ± 1 691
	NAD	21.2 ± 11.7	466 ± 115
	NADH	*bd*	1 572 ± 782
	NADP	3.1 ± 1.6	4.8 ± 1.6
	FAD	21.7 ± 4.5	4.1 ± 1.0
	PEP	*bd*	181 ± 110
Glycolysis/gluconeogenesis	Glucose	2 867 ± 757	36 081 ± 10 112
	Glucose 6-phosphate	82.1 ± 27.2	3 369 ± 2 600
	Fructose 1,6-bisphosphate	113 ± 52	4 022 ± 2 633
	Pyruvate	59.2 ± 28.7	245 ± 91.2
	Lactate	5 063 ± 1 076	60 111 ± 16 122
	α -ketoglutarate	9.3 ± 4.4	8.6 ± 5.2
TCA cycle	α-aminobutyrate	171 ± 71	1 424 ± 452
	Citrate	21.7 ± 8.9	173 ± 88.7
	Fumarate	207 ± 76	196 ± 72.9
	Cis-aconitate	0.0 ± 0.0	1.5 ± 0.5
	Malate	1 961 ± 758	1 863 ± 679
	Succinate	398 ± 191	2 869 ± 1 336
	FAD	21.7 ± 4.5	4.1 ± 1.0
Proteogenic amino acids	Alanine	3 283 ± 1 103	12 289 ± 3 457
	Aspartate	1 639 ± 544	588 ± 227
	Glutamate	8 500 ± 2 515	2 596 ± 736
	Glutamine	1 887 ± 1 129	2 769 ± 1 376
	Glycine	2 471 ± 680	10 401 ± 3 098
	Histidine	437 ± 226	4 781 ± 1 501
	Isoleucine	490 ± 203	542 ± 177
	Leucine	1 483 ± 647	1 082 ± 416
	Lysine	94.7 ± 22.3	83.6 ± 22.4
	Methionine	342 ± 154	280 ± 82.0
	Phenylalanine	550 ± 198	594 ± 186
	Proline	1 302 ± 440	1 738 ± 1 551
	Serine	2 060 ± 739	1 592 ± 808
	Threonine	1 401 ± 447	1 823 ± 632
	Tryptophan	93.7 ± 46.9	233 ± 65.8
	Tyrosine	505 ± 154	538 ± 151
	Valine	1 165 ± 417	1 103 ± 303
Other amino acids	α-aminoadipate	194 ± 79	64.0 ± 63.4
	Arginine	46.8 ± 21.4	78.9 ± 22.3
	β-alanine	568 ± 247	714 ± 395
	β-aminoisobutyrate	44.6 ± 12.1	75.7 ± 19.2
	Cystine	8.1 ± 4.3	126 ± 15.9
	Hydroxyproline	41.0 ± 25.6	1 010 ± 376
	Sarcosine	134 ± 79	742 ± 264
	Trimethylglycine	299 ± 236	1 672 ± 1 358

### Changes in metabolite concentrations

**Fig. 1:**
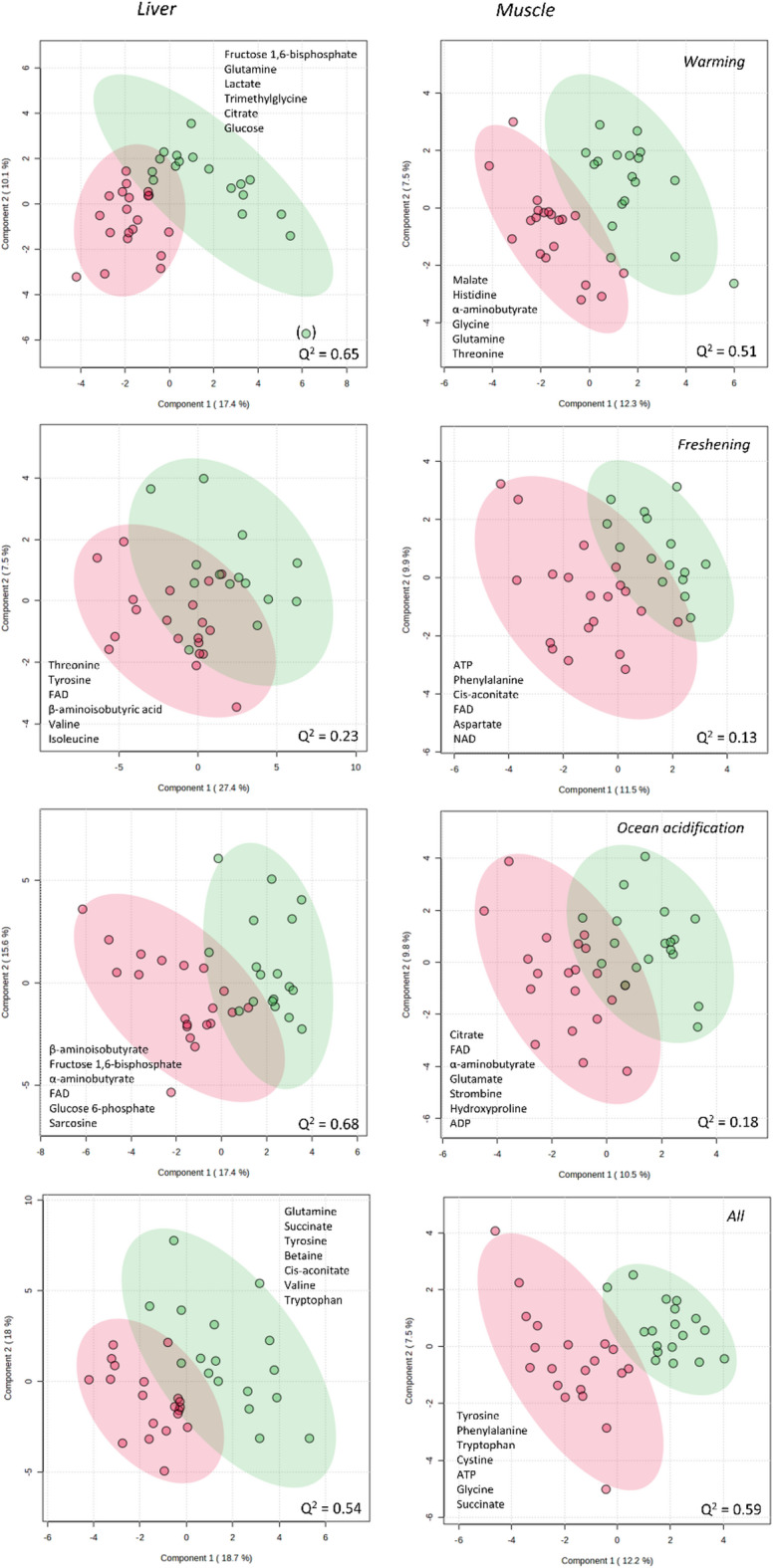
Partial least square discriminant analysis (PLS-DA) analyses of the overall structure of metabolite concentrations in samples within and between the single treatments (green) and the control (red). Most important metabolites contributing to the difference between treatment and control are indicated. Sample contained within parentheses was treated as an outlier and removed from the MetPA analysis. Values of Q^2^ toward one indicate increasing predictive ability of the PLS-DA model.

The changes in metabolite concentrations induced high predictability in both liver and muscle tissue in all treatments (PLS-DA: Q^2^ > 0.5), except for cod subjected to freshening (both tissues) and muscle samples of cod exposed to ocean acidification (Fig. 1).

**Fig. 2:**
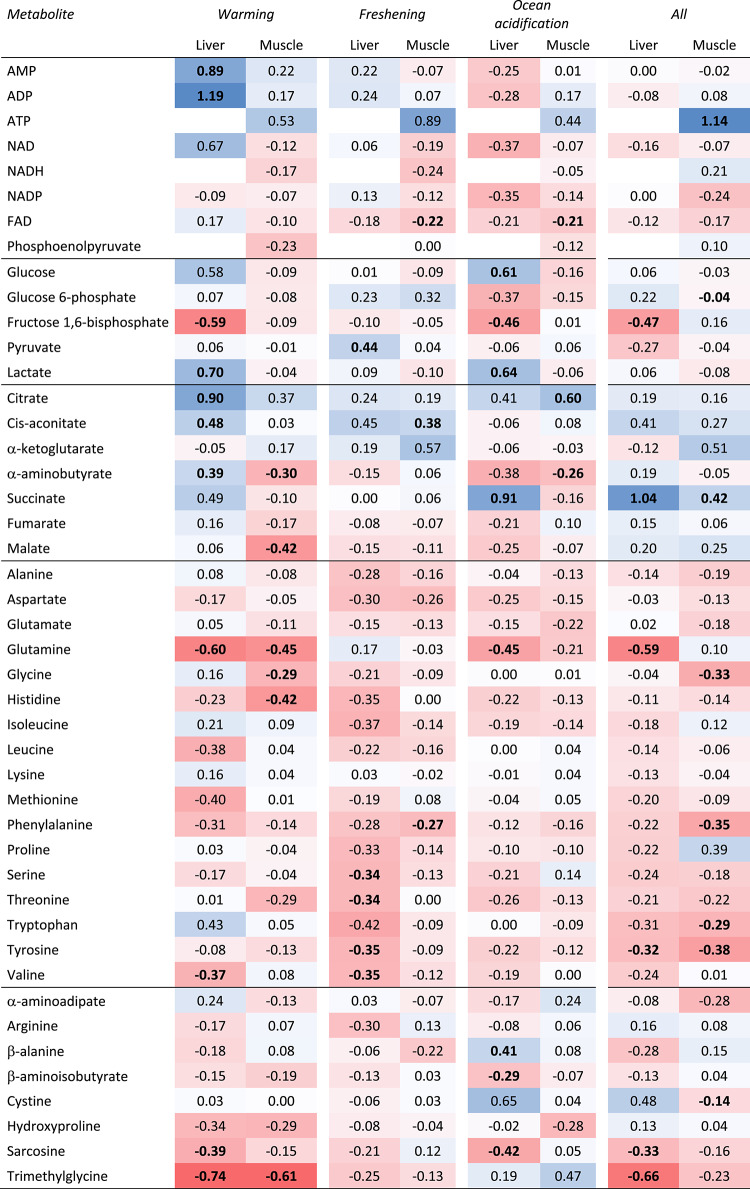
Differences in mean metabolite concentrations in liver and muscle tissue between treatments and the corresponding controls. Values depict fractional differences (a values of one equals a 100% increase). Colours reflect the values, red is negative (down-regulation) and blue is positive (up-regulation). The metabolites are grouped (horizontal lines) according to metabolic function, from the top down: Metabolites functioning as carriers of energy or reducing power, metabolites in the glycolysis/gluconeogenesis, metabolites in the TCA cycle, FAD, proteogenic amino acids, non-proteogenic amino acids. Values in bold show significant differences from the control (Dunnett’s test).

Warming induced up-regulation of most metabolites carrying energy or reducing power and metabolites involved in the glycolysis and the TCA cycle in the liver (Fig. 2) with the noticeable difference of fructose 1,6-bisphosphate, which was significantly down-regulated (ANOVA with Dunnett’s test: *P* < 0.05). The multivariate tests showed significant changes in the metabolite concentrations in the glycolysis (PERMANOVA pair-wise tests: P_perm_ = 0.001). In the muscles, changes were minor in these metabolites. In the TCA cycle, citrate, cis-aconitate and α-aminobutyrate were significantly upregulated (ANOVA with Dunnett’s test: *P* < 0.05). Surprisingly, these changes were not detected by the PERMANOVA multivariate test. There was no clear pattern of change in liver concentrations of proteogenic amino acids. However, both glutamine and valine were significantly up-regulated (ANOVA with Dunnett’s test: *P* < 0.05) and the multivariate test showed a significant change in proteogenic amino acids (PERMANOVA pair-wise tests: P_perm_ = 0.002). In the muscles, the two TCA cycle metabolites α-aminobutyrate and malate were downregulated and there was a significant overall change in metabolite concentrations (including those carrying energy or reducing power) (PERMANOVA pair-wise tests: P_perm_ = 0.035). Most amino acids were down-regulated, glutamine, glycine, and histidine significantly so (ANOVA with Dunnett’s test: *P* < 0.05), and there was a significant overall change in the proteogenic amino acids (PERMANOVA pair-wise tests: P_perm_ = 0.001). Of the non-proteogenic amino acids, trimethylglycine was significantly down-regulated both in the liver and muscles, and sarcosine was significantly down-regulated in the liver (ANOVA with Dunnett’s tests: *P* < 0.05).

Freshening induced a pattern similar to warming in liver metabolites involved in energy, glycolysis and the TCA cycle, albeit less pronounced, and only pyruvate was significantly up-regulated (ANOVA with Dunnett’s test: *P* < 0.05) (Fig. 2). In the muscles, the pattern was also similar to warming, and the multivariate test showed a significant change in the TCA cycle (including those carrying energy or reducing power) (PERMANOVA pair-wise tests: P_perm_ = 0.005). Amino acids were consistently down-regulated in the liver but less so in the muscles, and there was a significant change in the overall concentration pattern of proteogenic amino acids in the liver (PERMANOVA pair-wise tests: P_perm_ = 0.002). There were no significant changes in the non-proteogenic amino acids in the liver nor in the muscles.

OA induced down-regulation of all metabolites carrying energy or reducing power in the liver, although none of the changes were significant (Fig. 2). In the glycolysis, glucose and lactate were significantly up-regulated while fructose 1,6-bisphosphate was significantly down-regulated (ANOVA with Dunnett’s test: *P* < 0.05). Accordingly, the multivariate test showed a significant change in glycolysis metabolites (PERMANOVA pair-wise tests: P_perm_ = 0.015). Metabolites in the TCA cycle were both up- and down-regulated, succinate up-regulated significantly (ANOVA with Dunnett’s test: *P* < 0.05), and accordingly the multivariate test showed a significant difference in the TCA cycle (including those carrying energy or reducing power) (PERMANOVA pair-wise tests: P_perm_ = 0.005). In the muscles, the pattern of change was less pronounced and similar to that of both warming and freshening with citrate being up-regulated significantly (ANOVA with Dunnett’s test: *P* < 0.05). However, α-aminobutyrate was significantly downregulated (ANOVA with Dunnett’s test: *P* < 0.05), but there was no multivariate response. All proteogenic amino acids in the liver were down-regulated, glutamine significantly so (ANOVA with Dunnett’s test: *P* < 0.05), and the multivariate test showed an overall significant difference (PERMANOVA pair-wise tests: P_perm_ = 0.005). Changes were less pronounced in the muscles and there were no significant differences to the control. Of the non-proteogenic amino acids, β-alanine was significantly up-regulated (ANOVA with Dunnett’s test: *P* < 0.05) and β-aminoisobutyrate and sarcosine were down-regulated in the liver.

The All treatment did not induce any changes in metabolites carrying energy or reducing power in the liver (Fig. 2). However, as with warming and OA, fructose 1,6-bisphosphate (in the glycolysis) was significantly down-regulated (ANOVA with Dunnett’s test: *P* < 0.05). All but one metabolite in the TCA cycle were up-regulated, succinate significantly so (ANOVA with Dunnett’s test: *P* < 0.05), and the multivariate test showed an overall change (PERMANOVA pair-wise test: P_perm_ = 0.003). In the muscles, ATP was significantly up-regulated (ANOVA with Dunnett’s test: *P* < 0.05), while glucose 6-phosphate was slightly but significantly down-regulated (ANOVA with Dunnett’s test: *P* < 0.05). The multivariate tests showed an overall change in the TCA cycle (PERMANOVA pair-wise test: P_perm_ = 0.001) and, as in the liver, succinate was significantly up-regulated (ANOVA with Dunnett’s test: *P* < 0.05). All but one proteogenic amino acid were down-regulated in the liver, glutamate significantly so (ANOVA with Dunnett’s test: *P* < 0.05), and the multivariate test showed an overall change (PERMANOVA pair-wise tests: P_perm_ = 0.004). In the muscles, three proteogenic amino acids were up-regulated, while all others were down-regulated, glycine, phenylalanine, tryptophan, and tyrosine significantly so (ANOVA with Dunnett’s test: *P* < 0.05), and accordingly the multivariate test showed a significant change (PERMANOVA pair-wise test: P_perm_ = 0.001). Of the non-proteogenic amino acids, trimethylglycine and sarcosine were significantly down-regulated in the liver (ANOVA with Dunnett’s test: *P* < 0.05), whereas cystine was significantly down-regulated in the muscles (ANOVA with Dunnett’s test: *P* < 0.05).

Figures S1 and S2 in the supporting information show concentrations of all metabolites in the two tissues in the five different treatments.

### Enriched pathways

The four treatments induced changes in many different metabolic pathways. The changes in the 49 analysed metabolites gave rise to changes in on average 14.5 in the liver and 6.75 pathways in the muscles of the metabolic pathways recognised by MetaboAnalyst.

**Fig. 3:**
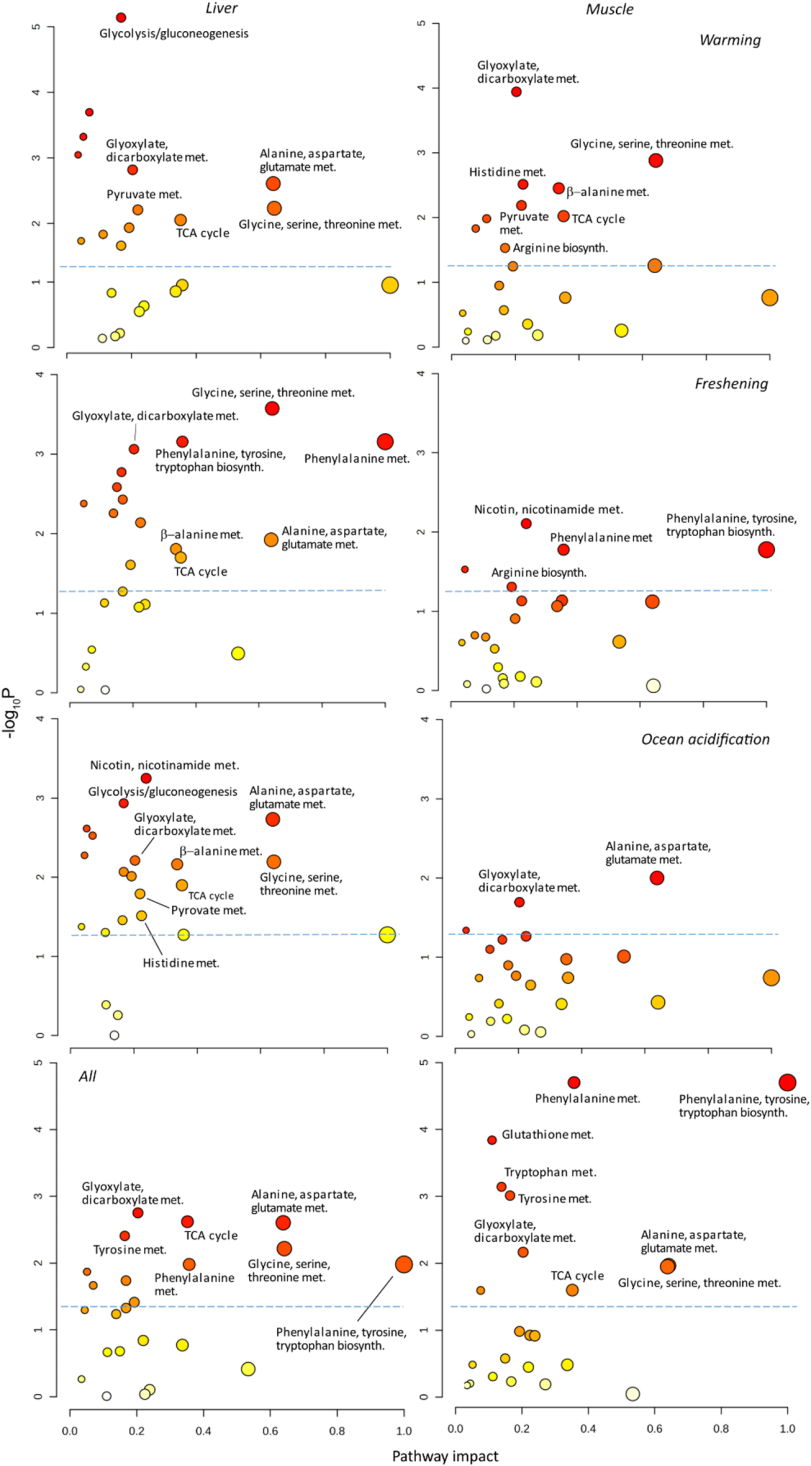
Results of the pathway analyses. Y-axes show the negative logarithm of the P value and X-axes show the impact of the tested molecules on the pathways. All pathways above the hatched blue lines are significantly enriched (*P* < 0.05). Thus, pathways in the top right diagonal region contain significantly enriched pathways in which the tested metabolites have important positions. Bubble colour vary according to –log_10_P and bubble size vary according to the pathway impact.

The changes in metabolites caused by warming were attributed to changes in the glycolysis or gluconeogenesis, but also in the TCA cycle and the metabolism of glyoxylate/dicarboxylate in the liver (Fig. 3). The glyoxylate/dicarboxylate metabolism is a variation of the TCA cycle largely absent from the metazoans and certainly absent in fish^33^. It is peculiar that this pathway should occur in an analysis based on the zebrafish metabolome and throughout the text we interpret it as representing the TCA cycle. Pyruvate metabolism was also affected, as well as the metabolism of a range of proteogenic amino acids. The TCA cycle and the pyruvate metabolism were also enriched in the muscles, along with the metabolism of a range of proteogenic amino acids. Moreover, the metabolism of β-alanine and arginine, two non-proteogenic amino acids were affected.

Freshening induced changes in the metabolism of several different amino acids and the biosynthesis of phenylalanine, tyrosine and tryptophan, along with changes in the TCA cycle (both the TCA cycle itself and glyoxylate/dicarboxylate metabolism) (Fig. 3). In the muscles, the metabolism and biosynthesis of several amino acids changed along with changes in the nicotine/nicotinamide metabolism.

Ocean acidification induced changes in the glycolysis, the TCA cycle and the pyruvate metabolism as well as changes in the metabolism of a range of proteogenic amino acids in the liver. Moreover, the nicotine/nicotinamide metabolism and β-alanine metabolism was enriched. In the muscles, the TCA cycle and the metabolism of the proteogenic amino acids alanine, aspartate and glutamate were enriched.

The All treatment induced changes in the TCA cycle and the metabolism of a range of proteogenic amino acids in the liver. The biosynthesis of phenylalanine, tyrosine and tryptophan was also enriched. In the muscles, the metabolism of a wide range proteogenic amino acids were strongly enriched as well as the biosynthesis of phenylalanine, tyrosine and tryptophan. The TCA cycle was also enriched.

## Discussion

In the present study, we identified both differences and similarities in the responses of cod to various climate-driven environmental stressors, namely warming, freshening, ocean acidification, and their combined effects. Overall, metabolic processes in the liver were more strongly affected than those in the muscle tissue. This finding is not unexpected, given that many regulating metabolic functions take place in the liver^34^. Due to their multifaceted function and involvement in such functions as energy production and storage, hormonal regulation, breakdown of toxins, osmoregulation and detoxification, processes in the liver will react to any changes in the external environment. Because of this, contrary to in other organs, transcription of new proteins and enzymes may increase more than 100-fold when fish are exposed to changes such as acute warming^35^.

The concentrations of all but a few amino acids decreased in both liver and muscles in response to the three stressors and their combination. The pathway analyses attributed these changes to metabolism of a range of different amino acids, and for all the different amino acid pathways available to the pathway analysis, only two pathways of biosynthesis of amino acids were significantly enriched, while ten pathways of metabolism of amino acids were significantly enriched across all the treatments. In fish, many organs (including the liver and muscles) possess enzymes to produce ATP, and thus energy, from amino acids in the TCA cycle^36,37^. Studies have shown that metabolism of glutamate, glutamine and leucine in the TCA cycle supply the most energy by far^38^. We observed concurrent changes in the TCA cycle, detected both as changes in concentrations of metabolites and as an enrichment of the TCA cycle in the pathway analyses. Also in European seabass (*Dicentrarchus labrax*) subjected to warming, metabolomic pathway analysis has suggested changes in glucose metabolism and the TCA cycle along with changes in a range of amino acids^39^.

The enrichment of glycine, serine and threonine metabolism in both liver and muscles and the significant decreases in trimethylglycine and sarcosine, both of which are constituents of glycine, serine and threonine metabolism, indicate that the metabolism of glycine, serine and threonine will increase in a climate change future. Along with increasing glutamate/glutamine metabolism and increasing muscular ATP concentrations in all treatments, these changes indicate that the environmental stress connected to climate change at a level predicted for the year 2100 will induce fundamental changes in amino acid metabolism in Atlantic cod, driven by the changes in all three environmental variables, although warming seems to induce the greatest changes. Overall decreases in amino acid concentrations have also been observed in the plasma in salmon (*Salmo salar*) after three months of a 6 °C increase in temperature^25^, and in zebrafish, warming induced elevated levels of metabolites associated with amino acid catabolism and lipid metabolism in the liver ^21^.

Besides their importance in energy production, both sarcosine and trimethylglycine partakes in osmoregulation^40^. However, we saw decreases in the concentrations of these amino acids in all treatments other than the freshening treatment. It therefore seems unlikely that the changes should be caused by osmoregulatory changes, rather the decreases are probably connected to changes in energy production.

The changes in liver concentrations of lactate, glucose and fructose 1,6-bisphosphate, along with the significant enrichment of the glycolysis/gluconeogenesis pathways in the liver is a strong indication of changes in the glycolysis or gluconeogenesis in cod exposed to warming and ocean acidification. A range of different experiments have established that fish, and especially carnivorous fish, do not depend on glucose for production of ATP but rather metabolises amino acids [reviewed in^41^]. Rather, glucose is metabolised through the pentose phosphate pathway for production of NADPH for lipid biosynthesis and ribose 5-phosphate required for nucleotide synthesis^42^. Lactate concentrations are naturally high in fish^43^ and any needed glucose is produced from lactate or pyruvate by gluconeogenesis. For this, the liver is an important site^44,45^, and studies have shown that there is a persistent high level of endogenous glucose production in the fish liver due to a lack of regulation of gluconeogenesis by dietary carbohydrates^42^. This production could be fuelled by the increased concentration of liver lactate we observed during both warming and ocean acidification. It seems that some process(es) in which glucose takes part are enhanced during warming and ocean acidification. In American shad (*Alosa sapidissima*), proteomic analysis showed that synthesis of enzymes involved in glycolysis/gluconeogenesis were significantly upregulated under warming in concert with increased metabolism of a range of amino acids (serine, isoleucine, cystine, choline and betaine)^46^. Perhaps increased production of enzymes to counter the stress induced by warming and ocean acidification in the cod could have triggered increased gluconeogenesis to supply nucleotide synthesis with ribose 5-phosphate. But enrichment in pathway analysis does not necessarily mean an upregulation. The amassed lactate and decreased fructose 1,6-bisphosphate concentrations under both warming and ocean acidification could indicate that gluconeogenesis was in fact downregulated through inhibition of step(s) in the chain of reactions between lactate and fructose 1,6-bisphosphate.

The significant increase in liver β-alanine concentrations during ocean acidification stands out as the only significant amino acid upregulation in both tissues in any treatment. Also, β-alanine synthesis was one of the two synthetic pathways enriched in the pathway analysis. Carnosine is a dipeptide, which is produced from β-alanine and histidine and acts as a buffer for the lactate produced during acidosis. The increased liver lactate concentrations under warming and ocean acidification could stem from such acidosis, and although we did not see any changes in histidine concentrations, it is possible that a further intensification of this cellular acidosis by extracellular decreasing pH from ocean acidification could have induced increased β-alanine demand for the synthesis of carnosine. Without decreased extracellular pH, the acidosis due to warming could have been countered without increased carnosine production.

Glutamate and glutamine constitute a large fraction of the amino acids in fish^47,48^ and we observed high concentrations of both in the cod. Unlike in mammals, glutamine does not serve as a nitrogen store for ammonium under normal conditions in fish, and circulating levels of this amino acid are therefore lower in fish^44^. Glutamate and glutamine are readily metabolised, and due to their high concentrations they are primary sources of energy in fish^36,47^, and together with aspartate and leucine they contribute as much as 80% of ATP production in the liver, intestines, kidney and skeletal muscles^38,49^. The significant enrichment of liver glutamate metabolism found by the pathway analysis in all four treatments accentuate the importance of the metabolism of glutamate and by extension glutamine also during future climate change stress. The molar contribution of glutamine to the total change in liver amino acids constituted 49%, 16% and 29% in the cod exposed to warming, ocean acidification and the All treatment, respectively. These significant decreases indicate that catabolism of glutamine in the liver will cover a large part of the increasing energy demand in cod during future climate change. Salmon (*Salmo salar*) has also been shown to experience this kind of change in glutamine plasma concentrations after three months incubation at a 6 °C temperature increase, so these alterations of energy production might be common in marine fishes in the future^25^.

To generate ATP in the TCA cycle, glutamine is converted to glutamate and the carbon backbone of glutamate is converted to α-ketoglutarate by glutamate dehydrogenase, a key enzyme in amino acid metabolism. Interestingly, glutamate dehydrogenase is activated allosterically by AMP and ADP but inhibited by ATP^50^, and we did find significantly increased concentrations of both AMP and ADP in the liver of cod exposed to warming supporting the notion that warming increases glutamine and glutamate metabolism in the liver. On the other hand, muscle ATP concentrations increased in all treatments, significantly so in the All treatment, so it may be that any catabolism of glutamine was limited to the liver. Alternatively, glutamine catabolism could also occur in the muscles, but it is perhaps first detectable after longer exposure.

Besides its importance in amino acid metabolism, glutamine also functions as a precursor in the synthesis of glutathione, an important inhibitor of oxidative stress^51^. Increased metabolism increases reactive oxygen species (ROS) production, and if the generation of ROS exceeds the capacity of defence, oxidative stress leads to severe cellular damage to proteins, lipids, and DNA^52^. Both shifts in temperature, salinity, and pH can lead to oxidative stress in aquatic animals including fish^53,54^. The enrichment of muscle glutathione metabolism in cod exposed to the All treatment could indicate that cod will experience these effects in a climate change future. Moreover, goldsinny wrasse from the same waters and subjected to similar treatments experienced significant increases in liver glutathione concentrations in the groups exposed to warming, freshening and a similar All treatment^55^, and in meagre (*Argyrosomus regius*), experiments with acidification and warming showed an increase in the activity of glutathione-S-transferase, an enzyme involved in glutathione’s action against oxidative stress, with warming^56^. On the other hand, Perry and colleagues^19^ found no significant changes in glutathione concentrations or the activity of glutathione-S-transferase or glutathione-reductase in the very same cod individuals from which we sampled for metabolomics. Moreover, the working of glutathione against oxidative stress depends on oxidation of NADPH to NADP^+^ and while NADPH concentrations were below detection in our experiment, we did not see any changes in NADP^+^ concentrations in any of the treatments.

Warming decreases cellular pH in fish^57^ and although acid-base regulation is accomplished mainly through regulation of cellular concentrations of HCO_3_^−^, excretion of NH_4_^+^ by the reduction of glutamine to glutamate could have contributed to acid-base regulation in the cod exposed to warming^58^. Thus, it is possible that increasing acidosis due to warming could have added to the catabolism of glutamine. This could also explain at least some of the decrease in glutamine in cod exposed to the ocean acidification treatment.

Glutamine is also used to supply nitrogen in the production of the nucleotides adenine and guanine. Thus, the demand for glutamine varies with the rate of transcription in the cells. It is therefore possible that the decreasing glutamine concentrations in cod exposed to warming, ocean acidification and the All environments could be attributed to increasing transcription to cover an overall increased enzyme demand due to stress. When glutamine act as a nitrogen donor in these pathways, glutamate is produced. However, liver glutamate concentrations did not increase significantly despite the decreases in glutamine. One simple explanation is of course that liver glutamate concentrations were so much higher (8500 nmol g^− 1^) compared to the glutamine concentrations (1887 nmol g^− 1^) that any changes in glutamate concentrations due to glutamine deamination disappeared in the variability among replicates. But, judging from the difference in mean concentrations between treatments and control, on average, 1130 nmol g^− 1^ glutamine was used and 446 nmol g^− 1^ glutamate produced during the warming treatment, while 1109 nmol mg^− 1^ glutamine was used and 166 nmol mg^− 1^ glutamate produced in the All treatment. We did not see a concomitant production of glutamate in the ocean acidification treatment, and while glutamine concentrations decreased by 844 nmol g^− 1^, glutamate concentrations also decreased, but by 1246 nmol g^− 1^. Thus, it seems that metabolism of liver glutamine primarily covered energy needs in the ocean acidification treatment (i.e. glutamate was converted to α-ketoglutarate in the TCA cycle at similar rates as glutamine was converted to glutamate), while during warming and in the All treatment, glutamine was primarily used to cover needs of other processes, such as nucleotide production for increased enzyme production. Consequently, while future ocean acidification will increase the natural glutamine/glutamate metabolism through the TCA cycle to cover increased energy demands, this natural upregulation of the TCA cycle will be overridden by the increased demand of nucleotides for increased overall enzyme production under future warming.

With the preconception that the effects of environmental stressors should be somewhat additive, one would expect that cod exposed to all three stressors would experience the most significant metabolic changes. We observed indications that it might not have been so and that warming and ocean acidification may have antagonistic effects in parts of the cod metabolism. Although amino acid metabolism was affected similarly by warming and ocean acidification, liver AMP and ADP concentrations behaved oppositely in these two treatments. AMP and ADP are produced when ATP delivers energy by oxidation, and changes in the concentration of AMP and ADP should indicate changes in the energy demand of the processes supplied by ATP. Moreover, NAD^+^ concentrations showed a similar opposite behaviour in the two treatments. NAD^+^ is a co-enzyme in cellular redox reactions with a central role in cellular energy production. The ability of NAD^+^ to accept a hydride ion, forming its reduced version, NADH, regulates the activity of dehydrogenases involved in many catabolic pathways, including glycolysis, glutaminolysis and fatty acid oxidation^59^. The accepted electrons in these reactions are then donated to the electron transport chain to form ATP. All these changes were absent from the liver in cod exposed to all three stressors in concert indicating antagonistic effects in the processes leading to the formation or transformation of these molecules. All biochemical processes increase with temperature^60^ but the effects of increasing acidity are more complex and may entail metabolic depression. Extracellular acidification can cause a shift in cellular pH regulation from energy demanding Na^+^/H^+^ exchange to the less energy demanding Na^+^/H^+^/Cl^−^/HCO_3_^−^ exchange to rid the body of excess HCO_3_^−^^61,62^, and studies on European flounder (*Platichthys flesus*) show that marine fishes may experience a reduction of the metabolic energy demand with decreasing pH^63^. The result of these two counteracting changes is that while the rate of aerobic performance and hence the organismal energy demand increase within the temperature pejus range until the maximum pejus temperature, beyond which it declines, increasing environmental pH will both depress overall performance and narrow the pejus range so that increasing energy demand due to warming and metabolic depression caused by ocean acidification may counteract each other within the thermal pejus range^64^. Although in the high end, the warming treatment temperature (18 °C) was within the thermal pejus range of cod^65,66^, so it is plausible that the cod did in fact experience antagonistic effects from warming and ocean acidification on the demand for ATP.

The significant increase in succinate liver concentrations in cod exposed to ocean acidification and liver and muscle concentrations in the All treatment stand out from the otherwise more general decrease in TCA metabolites in these treatments. This is rather unlike under warming where all but one liver metabolite increased. The increases under ocean acidification and the All treatment could indicate a reduced activity of succinate dehydrogenase (SDH). SDH is placed in the inner mitochondrial membrane and is the only enzyme to function in both the TCA cycle and the oxidative phosphorylation^67^. SDH transfers electrons from the TCA cycle to the oxidative phosphorylation through the reduction of FAD. However, we did not see any concomitant significant changes in FAD concentrations. Besides its function in the TCA cycle and oxidative phosphorylation, succinate is also involved in the formation and removal of reactive oxygen species in the mitochondria and is thus important for oxidative stress mediation^68^. Perhaps alleviation of oxidative stress caused the noteworthy increase in succinate during ocean acidification and the All treatment. Since there were no concomitant changes in succinate in the muscles, the significant decreases of FAD during freshening and ocean acidification were probably not caused by an increased FAD demand coupled to the TCA cycle or oxidative phosphorylation. However, FAD is a multifaceted co-enzyme functioning in a large variety of metabolic pathways including electron transport, DNA repair, nucleotide biosynthesis, beta-oxidation of fatty acids, amino acid catabolism, as well as synthesis of other cofactors such as CoA, CoQ and heme groups^59^. Obviously, decreasing concentrations of FAD will influence these functions, but we cannot say which and to what extent.

Biosynthesis of phenylalanine, tyrosine and tryptophan were the only enriched biosynthetic pathways. These aromatic amino acids are precursors of stress hormones such as dopamine and norepinephrine ^69^ and increased biosynthesis may indicate a response to alleviate stress. Accordingly, supplementing the diet with these amino acids have shown to relieve farmed fish from stress symptoms^69^. Because we observed this enrichment only in the freshening and All treatments, it may be hypothesised that the biosynthesis of these amino acids were augmented to meet osmotic stress. Very little information to support this hypothesis exists, but in the Amur ide (*Leuciscus waleckii*), this biosynthetic pathway, among others, was enriched when subjecting the fish to osmotic stress^70^. This species is, however, adapted to hyperosmotic living conditions and may not be a good representative for osmoregulation in other teleost fish.

Exposure to changes in environmental conditions, such as those caused by global climate change, can lead to stress in marine organisms such as fish. This, in turn elicits a physiological response in the fish, which is thought to be an adaptive strategy to maintain homeostasis. However, if the stress is too severe or long lasting the fish may be unable to regain homeostasis leading to a maladaptive physiological response, which can ultimately result in negative consequences for individuals’ health. As we show here, the metabolic costs associated with exposure to climate change stress are both multi-facetted and profound, and there is no doubt that under chronic exposure these changes will have consequences for individual health. While stress response is individual, when environmental changes occur on large scales, entire populations are subjected to the same stressors leading to potential negative consequences for entire fish populations. In the case of Atlantic cod who’s populations already have extremely poor status in northern European waters^15,16,71,72^, the metabolomic changes we show here certainly are concerning.

## Supplementary Information

Below is the link to the electronic supplementary material.

Supplementary Material 1

## Data Availability

The datasets used and/or analysed during the current study are available from the corresponding author on reasonable request.
