# Supplementary material for: Impact of climate change driven freshening, warming, and ocean acidification on the cellular metabolism of Atlantic Cod (Gadus morhua)
Source: Sci Rep. 2025 Oct 23;15:37155. doi: 10.1038/s41598-025-21597-z (PMC12550006; doi:10.1038/s41598-025-21597-z)

Figure S1. Liver samples. Metabolite concentrations after outlier removal and imputations. C is control, W is the warming treatment, F is the freshening, OA is the ocean acidification treatment, and All is the multi-stressor treatment.

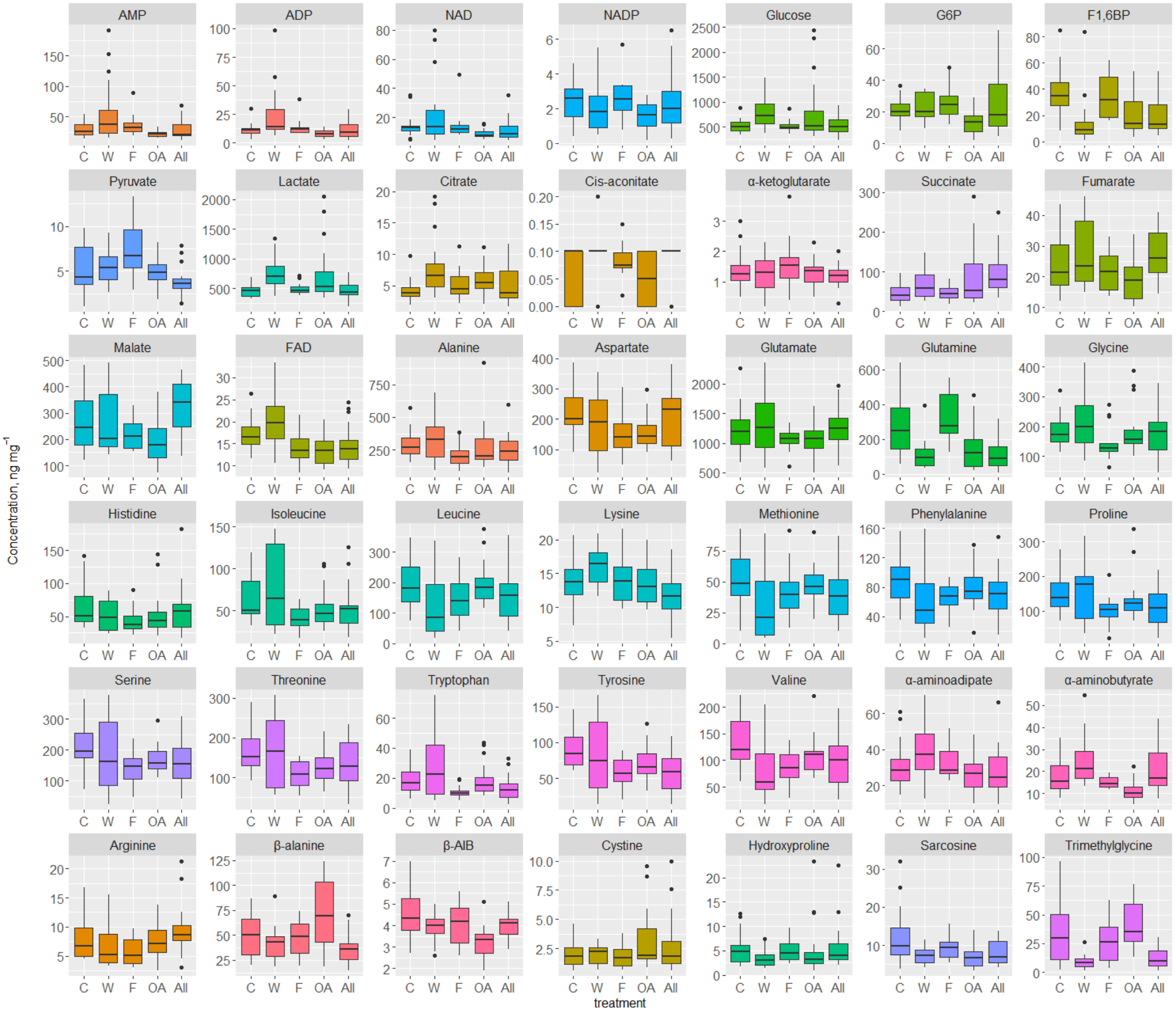

Figure S2. Muscle samples. Metabolite concentrations after outlier removal and imputations. C is control, W is the warming treatment, F is the freshening, OA is the ocean acidification treatment, and All is the multi-stressor treatment.

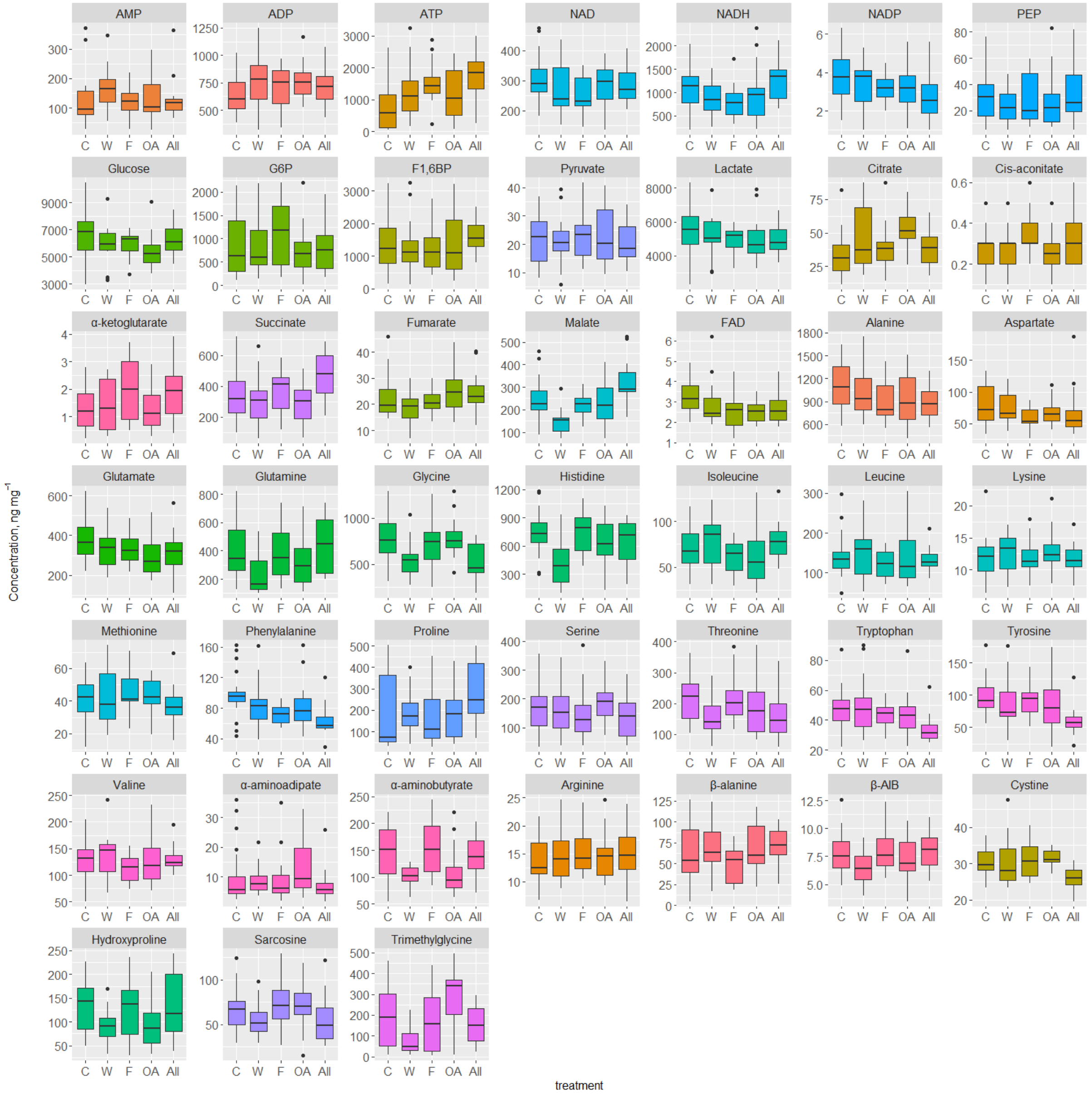

Supplement: Supplementary file 1 — Supplementary Material 1 [file 41598_2025_21597_MOESM1_ESM.pdf]
